# Treatment of Mucous Retention Cyst in Association with Sinus Lift and Implant Placement: A Case Report with 1-Year Follow-Up

**DOI:** 10.1155/2023/6968487

**Published:** 2023-09-14

**Authors:** Antoine Berberi, Georges Aad, Sara Kebbe, Rebecca El Hachem, Nabih Nader

**Affiliations:** ^1^Department of Oral and Maxillofacial Surgery, Faculty of Dental Medicine, Lebanese University, Beirut, Lebanon; ^2^Department of Oral Medicine and Maxillofacial Radiology, Faculty of Dental Medicine, Lebanese University, Beirut, Lebanon

## Abstract

Sinus lift augmentation techniques, lateral or crestal approaches, have been well documented, with bone substitute graft, or without bone material, with immediate or delayed implant placement as a treatment option for the atrophic maxilla in the posterior area. However, the sinus lift procedures performed in the presence of cysts, mucoceles, mucous retention cysts (MRCs), and antral pseudo-cysts could mainly decrease the sinus cavity volume and could increase the possibility of ostium obstruction and might lead to infection followed by failure of the grafting procedure. A radiological assessment should be made with computerized tomography (CT) or cone-beam CT to evaluate the remaining bone volume and to detect any pathology in the sinus. Different techniques were described in the literature for sinus lifting and bone grafting in patients with cysts. For some authors, cysts should be treated before sinus grafting and six months later, the procedure could be performed. For others, sinus lifting can be performed without lesion removal. At this time, controversy exists regarding the decision on whether lesions must be removed/aspirated or not before sinus grafting. In this study, we report a case where an MRC was aspirated and instantaneously, the sinus membrane was lifted and grafted, and implants were installed with 1-year follow-up after loading. Identifying lesions in the maxillary sinus is essential before planning any type of sinus augmentation and implant placement.

## 1. Introduction

The treatment of the atrophic posterior maxilla with sinus lift augmentation techniques has been well-documented with a high rate of success [1, 2].

The remaining height of the crestal bone orients the practitioner for lateral or crestal approaches with bone substitute graft [3–5] or without bone material [3, 6] with immediate [1, 2] or delayed implant placement [2, 7].

However, the sinus lift procedures when performed in the presence of cysts could remarkably decrease the sinus cavity volume, increase the possibility of ostium obstruction, and might lead to sinusitis followed by failure of the grafting [8, 9].

These cysts incorporate mucoceles (M), mucous retention cysts (MRCs), and antral-pseudo cysts (APCs) [10].

Thus, the sinus anatomy and its Schneiderian membrane should be carefully evaluated when a sinus lift procedure is planned [11, 12].

MRCs and APCs are frequently found incidentally during radiographic examinations [10].

MRCs are frequent lesions and appear after a high proliferation of the fluid's level inside the sinus membrane and manifest as dome-shaped radiopacities in the sinus [13, 14].

Although its etiology is controversial, many investigators have suggested an environmental cause and their formation has been related to seasonal changes, mainly in the beginning of spring and autumn [13, 15].

Most of them are asymptomatic, although some discomfort such as congestion, postnasal drip, flow of yellow fluid from the nose, headache, and recurrent rhino sinusitis that could exceptionally result in nasal obstruction may be reported [14, 16].

Cysts can dissolve without any medication. In 60% of cases the volume remains stable, in 30% the volume shrinks or can disappear completely, and only in 10% the volume increases [13].

Bhattacharyya found that MRCs are found in one sinus in 12.4% of cases and both sinuses in 18% of cases. In 50% of cases, they were located on the sinus floor. In 88% they were solitary [17]. Shear and Speight revealed that the frequency rate of APCs varies between 1.6% and 8.7% [18].

Wang et al. described that most MRCs of the maxillary sinus suddenly relapse or show a non-significant change in volume [16].

A differential diagnosis should be made with other lesions, such as mucoceles and inverted papilloma [12, 13, 15].

Implant placement in the atrophied posterior region of the maxilla needs precision and evaluation of the maxillary sinus. The panoramic radiographs provide a general appreciation, but it is not suitable for a complete assessment [19].

A precise evaluation should be done with computerized tomography or cone-beam computed tomography (CBCT) to determine the remaining bone volume and to detect any pathology in the sinus to be able to plan the maxillary sinus augmentation procedure with or without simultaneous implant placement [19, 20].

Different techniques were described in the literature for sinus lifting and bone grafting in patients with MRC and APC [8, 9, 21]. Some authors reflected their existence as a contraindication and suggested a previous lesion removal before grafting [22]. Six months later, the sinus grafting could be performed and implants could be placed at the same time or postponed for a period of 3 months to achieve an osseointegration [23].

For other authors, patients could undergo sinus lifting with no lesion removal, in a single stage or delayed approach [24].

In this study, we report a case where an MRC was aspirated, instantaneously, the sinus membrane was lifted, and implants were placed.

## 2. Case Report

A 47-year-old female patient visited the Department of Oral & Maxillofacial Surgery, complaining of masticatory difficulties due to the loss of her left maxillary posterior teeth besides her need for a sinus lift to be able to place the implants. The interrogatory revealed that the patient had no medical problems.

**Figure 1:**
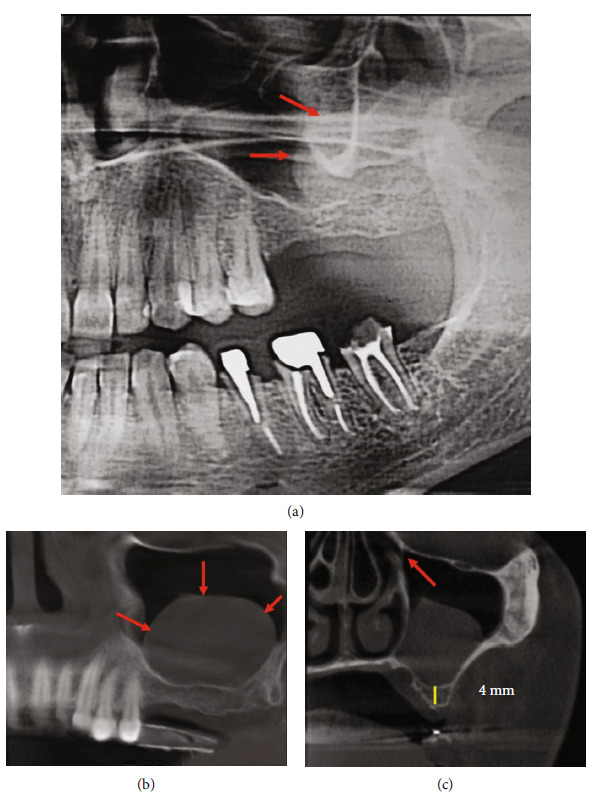
(a) Panoramic radiograph showed a radiopacity in the left maxillary sinus (arrow: border of the cyst). (b) A sagittal cut of the CBCT displayed a round-shaped radiopaque lesion on the left maxillary sinus (arrow: border of the cyst). (c) Axial cut revealed the radiopacity lesion inside the sinus, 4 mm residual bone height, and the ostium remains clear (arrow: ostium).

A panoramic radiograph showed a missing first and second maxillary molars with a residual bone height of less than 4 mm, and a radio-opacity image was observed in the left maxillary sinus (Figure 1(a)). CBCT revealed a round-shaped radio-opaque lesion on the left maxillary sinus with a clear ostium (Figures 1(b), 1(c) and 1(d)).

**Figure 2:**
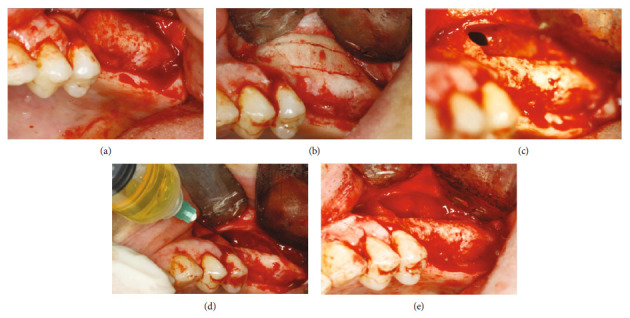
(a) Mucoperiosteal flap was elevated, and the bone wall of the sinus was exposed. (b) Osteotomies of the bony window were performed with the piezo surgery. (c) The bony window was detached in one piece, and a small perforation of the sinus membrane was observed in the upper mesial part of the window. (d) The yellow cystic fluid was aspirated with a sterile syringe with a 22 G needle. (e) The Schneiderian membrane was lifted carefully through the bony window area, and the perforation was sealed with a collagen membrane.

The preliminary diagnosis varies between an antral pseudo-cyst or MRC. The treatment plan was to aspirate cystic fluid to lift the sinus membrane to place the two implants simultaneously, and restore the missing molars. The surgical procedure was performed under local analgesia (4% articaine with 1 : 200,000 epinephrine). A crestal incision was made medially from the second premolar to the third molar area with two lateral releasing incisions. Then, a muco-periosteal flap was elevated and the bone wall of the sinus was exposed (Figure 2(a)). Osteotomy of the bony window was done with the piezosurgery instruments. The bony window was removed in one piece, and then the sinus membrane was lifted by a special curette and raised to achieve a curtain effect (Figure 2(b)).

A small perforation of the sinus membrane was observed in the upper mesial part of the window (Figure 2(c)). The cystic fluid was aspirated through the perforation with a sterile syringe with a 22 G needle (volume 5 ml). The aspirated fluid color was yellow, and the cyst membrane was sent for histological evaluation (Figure 2(d)).

The Schneiderian membrane was then lifted carefully through the bony window area, and the perforation was sealed with a collagen membrane (CollaTape^®^ Zimmer-Biomet; Figure 2(e)).

**Figure 3:**
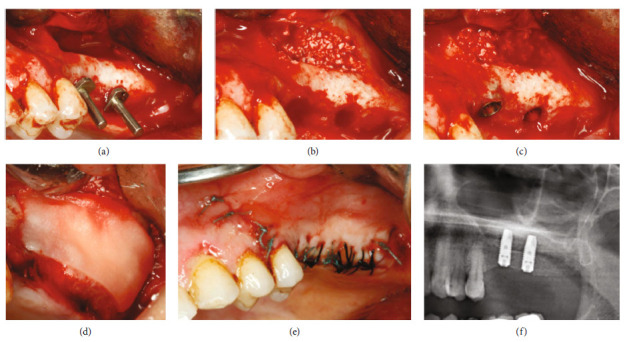
(a) Implant sites were prepared. (b) The first layer of bone substitute was packed into the cavity. (c) 2 implants were placed, and the final layer of bone substitute was placed. (d) Resorbable membrane was positioned under the bony wall. (e) Interrupted O sutures were made using a 3-0 silk suture. (f) Panoramic radiograph showed a well-defined area with two implants in the middle.

Implant sites were drilled, and the first layer of bone substitute was packed into the cavity between the residual crestal bone, the palatal bone, and the Schneiderian membrane (Figures 3(a) and 3(b); Puros Cortical 0.25–1.0 mm particulate; Zimmer Biomet). Two implants (4 mm × 13 mm, Astra Tech-Dentsply^®^) were then placed. The final layer of bone substitute was placed (Figure 3(c)) and a resorbable membrane was positioned under the bony wall (Figure 3(d)). Interrupted O sutures were made using a 3-0 silk suture (Figure 3(e)).

Post-operative medications based on amoxicillin–clavulanic acid 1 g as an antibiotic (2 g/day for 7 days), mefenamic acid as anti-inflammatory (two tablets/day for 5 days), paracetamol, codeine phosphate hemihydrate, and caffeine in combination as painkillers (two tablets in case of pain), 2-week prescribed mometasone furoate as a systemic nasal decongestant (twice/day), and 0.12% chlorhexidine gluconate as an oral rinse (three times/day with water for 10 days). One week later, the sutures were removed, and no signs of nasal congestion were stated.

**Figure 4:**
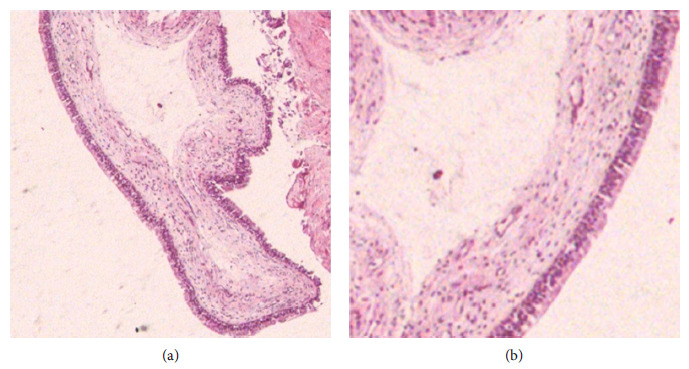
(a, b) The histological sections showed a virtual cystic cavity lined by a columnar pseudo-stratified epithelium with an underlying layer of loose connective tissue and a mononuclear inflammatory infiltrate was noted in the underlining tissue (H&E ×20 and H&E ×40).

The microscopic result after cyto-centrifugation came from some blood cells with macrophages and inflammatory cells, and the diagnosis was in favor of MRC. In addition, the histological sections showed a virtual cystic cavity lined by a columnar pseudo-stratified epithelium with an underlying layer of loose connective tissue, and a mononuclear inflammatory infiltrate was noted in the underlining tissue [hematoxylin and eosin (H&E) ×20 and H&E ×40)] (Figures 4(a) and 4(b)).

Panoramic radiograph showed a well-defined area with two implants in the middle (Figure 3(f)).

**Figure 5:**
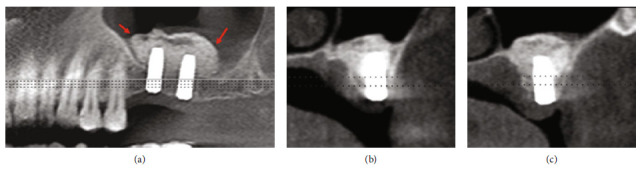
(a) Panoramic reconstruction showing well-limited grafted material within the middle of the two implants. No pathology was detected in the sinus. (b) The para-axial cut of the mesial implant completely embedded in the graft material. (c) The para-axial cut of the distal implant also well surrounded by the bone graft.

**Figure 6:**
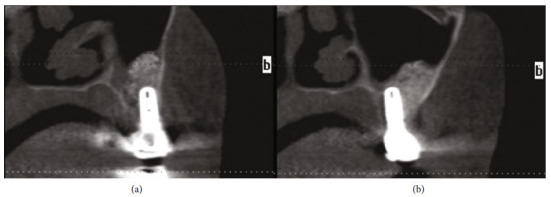
Axial cut of the left maxilla, one year after loading, showing the implant surrounded by the grafted material without any recurrence of the mucous retention cyst. (a) The first implant. (b) The second implant.

The panoramic reconstruction and para-axial cuts of the CBCT at 5 months showed a well-limited augmented area covering from the buccal to the palatal bone, and no pathologies were detected inside the maxillary sinus (Figures 5(a), 5(b), and 5(c)). Crowns were fabricated and cemented to the abutments. The para-axial cut of the CBCT one year after loading showed a very integrated graft inside the sinus, and the implants are well integrated (Figures 6(a) and 6(b)).

## 3. Discussion

Disagreements exist about the indications of the sinus lifting procedure when MRC and APC are present, without previous treatment and a waiting period for healing achievement [10, 11].

Treatment of those lesions during the sinus augmentation procedure by aspiration or surgical excision is very well documented in the literature and some authors state that decreasing the cyst volume by aspiration helps to reduce the intra-sinus pressure and thus, the risk of perforation of the Schneiderian membrane [10, 11, 23, 24, 25].

For others, when the lesions are symptomatic or the diagnosis is uncertain, enucleation should be considered before sinus lift surgery [23, 26].

Treatment planning, when sinus lift surgery should be performed and when cysts exist inside the maxillary sinuses, is divided into three options:

1. Cyst/pseudocyst should be treated before the sinus lifting procedure and implant placement [25, 26].
2. Aspiration/removal could be performed simultaneously with sinus graft surgery [27–29].
3. The lesion can be left untreated [26, 28, 29].

Schneiderian membrane perforation is one of the common accidents that occur in sinus grafting with a variable rate between 7% and 60% [1, 30].

Moreno Vazquez et al. in a retrospective study of sinus lifting evaluating 127 patients, reported a high rate of Schneiderian membrane perforation in 25.7% [31].

Díaz-Olivares et al. in a systematic review that included 1,598 sinuses lifting using the lateral approach, reported a perforation rate of 30.6% [32].

Small or limited perforations are corrected intra-operatively by using collagen membranes [33] two separate bioabsorbable membranes [34], or fibrin glue that could lead to a newly formed epithelium [35] or PRF for its autogenous characteristics [36].

Park et al. recommend that the blood clot after perforation leads to membrane repair [37]. Testori et al. suggested that self-repairing could be observed with small perforations [38].

Large perforations could be treated with a two-stage approach using a collagen sponge [39] or by suturing the membrane with resorbable sutures [36, 40]. In those cases, the augmentation surgery will be postponed from 3 to 6 months to permit membrane reparation [41].

In our case, the liquid of the cyst was aspirated, and the small perforation was treated with collagen membrane during the sinus lift procedure and the implants could be placed with a good primary stability.

This technique reduced the treatment period and the patient recuperated her missing teeth in a short period.

## 4. Conclusion

In conclusion, the aspiration of the cyst fluid concomitantly with the sinus lift procedure with bone substitutes shows a new bone formation inside the sinus and around the implants according to radiographic and clinical assessments. Identifying lesions in the maxillary sinus is essential before planning any type of sinus augmentation and implant placement. Do we need to remove the cyst before or during the sinus lifting procedure?

Each case should be assessed individually and a discussion with an Ear–Nose–Throat specialist could be required. Since in our case, the cyst was small without any obstruction and ventilatory problems, aspiration and sinus augmentation simultaneously reduced the number of surgeries and shortened the treatment time. The small perforation treated with collagen and bone substitute was well covered by the sinus membrane.

## Data Availability

The available data are presented in the manuscript.
